# Burkitt-Like Lymphoma with 11q Aberration: A Case Report and Review of a Rare Entity

**DOI:** 10.1155/2020/8896322

**Published:** 2020-09-07

**Authors:** Sepideh N. Asadbeigi, Chelsey D. Deel

**Affiliations:** Department of Pathology, University of Oklahoma Health Sciences Center, Oklahoma, USA

## Abstract

Burkitt-like lymphoma with 11q aberration is a rare diagnostic entity commonly occurring in children and young adults with a nodal presentation. This entity shares many similar morphologic and immunophenotypic features with conventional Burkitt lymphoma and other aggressive B-cell lymphomas, making its recognition challenging. However, the presence of its characteristic 11q gain/loss pattern is helpful in the diagnosis. We report a case of Burkitt-like lymphoma presenting as a right neck mass in a 17-year-old female patient that demonstrated no improvement with antibiotic therapy. The neoplasm displayed a diffuse proliferation of intermediate-sized atypical lymphoid cells with prominent nucleoli in a background of apoptotic debris, morphologically raising concern for conventional Burkitt lymphoma. Subsequent immunohistochemical and cytogenetic studies established the most likely diagnosis of Burkitt-like lymphoma with 11q aberration. Though rare, Burkitt-like lymphoma exhibits significant morphologic overlap with other high-grade B-cell lymphomas, making it an important entity to consider on the differential diagnosis of these lesions.

## 1. Introduction

Burkitt lymphoma (BL) is a well-defined aggressive B-cell lymphoma with rearrangements characteristically involving MYC and the immunoglobulin (IG) heavy (t(8;14)) or, more rarely, light chain loci (t(2;8) and t(8;22)) [1, 2]. The term Burkitt‐like lymphoma with 11q (BLL-11q) aberration has emerged as a new provisional diagnostic entity in the Revised 4th Edition of the World Health Organization (WHO) Classification of Tumors of Hematopoietic and Lymphoid Tissues to describe cases morphologically resembling BL that have unique clinical, cytogenetic, and molecular features [3]. Initially reported by Poirel and colleagues in 2009 [4], this lymphoma is a rare and aggressive lymphoid neoplasm which is defined by the genetic hallmark of its 11q-gain/loss pattern. BLL-11q morphologically resembles Burkitt lymphoma (i.e., “starry sky” pattern and high proliferative rate) and has a similar immunophenotype (CD10+, BCL-6+, BCL-2(-), and Ki-67 approaching 100%) [1, 2, 5]. However, this lymphoma lacks *MYC* rearrangements and carries an 11q-arm aberration with proximal gains and telomeric losses [2]. Because of its resemblance to other aggressive B-cell lymphomas such as Burkitt lymphoma and unique associated clinical/prognostic features, Burkitt-like lymphoma with 11q aberration remains an important diagnostic entity for consideration in these lesions.

## 2. Case Report

A 17-year-old previously healthy female presented to the outpatient surgery clinic for evaluation of a right neck mass, present for eight months and demonstrating no improvement with multiple rounds of antibiotic therapy. The patient had no relevant personal or family history of malignancy and denied B symptoms. Physical examination identified a large right substernocleidomastoid nontender and immobile mass. A computed tomography (CT) scan in January 2019 revealed an enlarged lymph node within the right jugular digastric region (3.0 cm in greatest dimension), causing mass effect on the jugular vein. A repeat CT scan performed in August 2019 demonstrated interval increase in the size of the lymph node (7.3 × 5.6 × 3.5 cm). The patient was subsequently referred for surgical biopsy of the mass.

**Figure 1:**
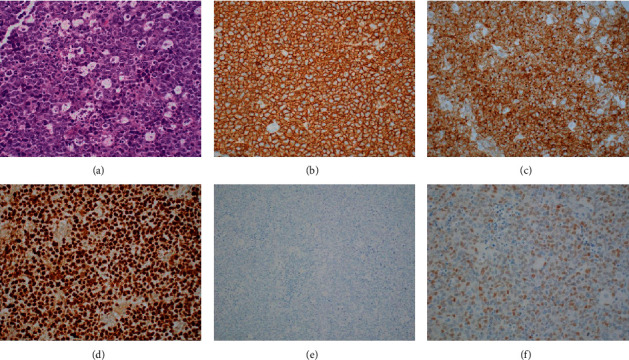
Neck mass in a 17-year-old female. (a) Sections show a diffuse proliferation of intermediate-sized atypical cells with prominent nucleoli in a background of apoptotic debris and numerous tingible-body macrophages (hematoxylin-eosin, original magnification 40x). The tumor cells are positive for (b) CD20 (original magnification 40x), (c) CD10 (original magnification 40x), and (d) BCL-6 (original magnification 40x) and negative for (e) CD56 (original magnification 20x). The tumor cells show weak *MYC* expression (f) (original magnification 40x).

**Figure 2:**
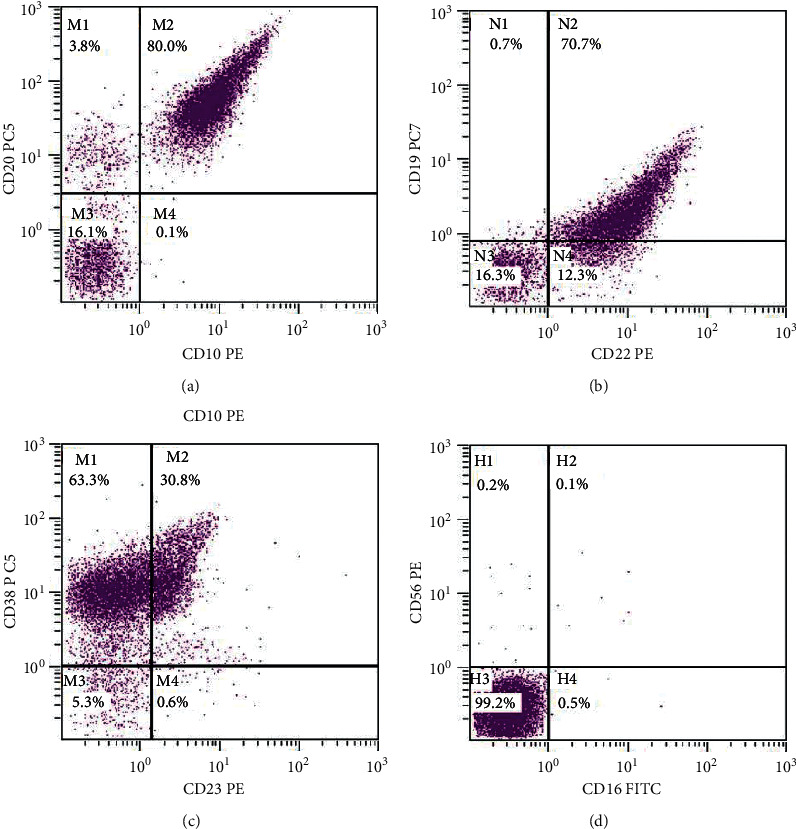
Flow cytometry from a neck mass in a 17-year-old female demonstrating neoplastic (a) CD20-positive B-cells with CD10 co-expression. The neoplastic cells demonstrate positive expression for (b) CD19, CD22, and (c) CD38 with (d) no CD16 or CD56 expression.

Frozen section assessment of the cervical mass was suggestive of Burkitt lymphoma. Microscopic examination of the resected tissue showed a diffuse proliferation of intermediate-sized atypical lymphoid cells with prominent nucleoli in a background of apoptotic debris. By immunohistochemistry, the high-grade cells were positive for CD20, CD10, BCL-6, and MYC and negative for BCL-2, TdT, CD30, CD56, and MUM-1. Ki-67 highlighted a high proliferative index (Figure 1). Flow cytometry identified 86% of events as lymphocytes, a majority of which were B cells expressing CD45 (bright), CD20, CD19, CD10, CD22, CD38 (moderate-bright), FMC-7, CD23 (dim partial), and kappa light chain (Figure 2). No expression of CD56 was identified. Based on morphology, a differential diagnosis of Burkitt lymphoma, diffuse large B-cell lymphoma, and high-grade B-cell lymphoma was entertained.

Fluorescence in situ hybridization (FISH) testing with break-apart probes specific for rearrangements of the *BCL6* gene, *MYC* gene, *BCL2* gene, and t(8;14) did not show any abnormal rearrangements. Conventional cytogenetics identified two abnormal cell lines. The first abnormal cell line had a modal number of 46 chromosomes, including one normal *X* chromosome and attachment of unknown material to another *X* chromosome at p22.1 as well as chromosome 11 at q23. The second abnormal cell line had unknown material attached to 4q31.3 in addition to the anomalies detected in the first cell line. A staging bone marrow biopsy demonstrated no evidence of involvement by lymphoma. Overall, the clinical presentation, morphology, and concurrent ancillary studies make Burkitt-like lymphoma with 11q aberration the most likely diagnosis.

**Figure 3:**
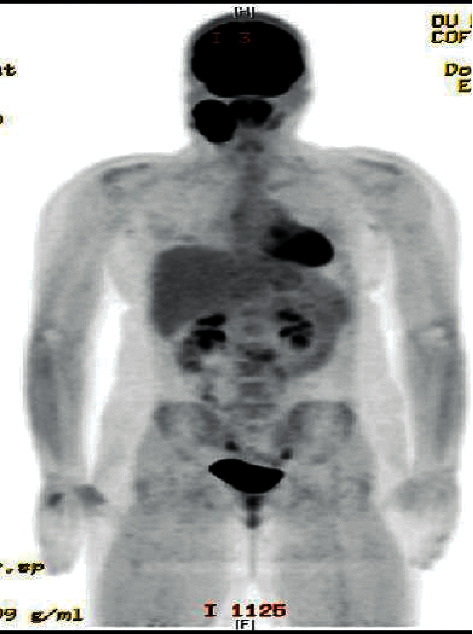
PET scan from a 17-year-old female showing a large FDG-avid right cervical mass, compatible with primary malignancy. No FDG-avid malignancy is identified elsewhere in the body.

A positron emission tomography (PET) scan identified hypermetabolic activity (SUV 34.1) in the primary right level 2A cervical mass (5.8 × 4.1 cm) as well as a left level 2A lymph node (1.6 × 1.1 cm, SUV 2.4) (Figure 3). The patient received treatment per ANHL 1131 group B protocol, including induction chemotherapy with cyclophosphamide, vincristine, prednisone, doxorubicin, and methotrexate (COPADM) and consolidation chemotherapy with cytarabine and methotrexate (CYM). The patient also received intrathecal methotrexate and hydrocortisone therapy. Follow-up CT scans showed a continuous significant reduction in the tumor size compared to prior analysis. A posttreatment PET/CT scan demonstrated a marked interval decrease in size and hypermetabolism of the right cervical mass, compatible with a near complete response. The patient is being followed per post chemotherapy protocol, and, to date, there is no evidence of new disease or recurrence.

## 3. Discussion

### 3.1. Clinical Features

Burkitt-like lymphoma with 11q aberration (BLL-11q) is a rare, aggressive lymphoid neoplasm, accounting for less than 10% of all Burkitt lymphoma cases in most series [2, 6]. Interestingly, its incidence may be enriched in the posttransplant setting, with one article reporting BLL-11q in 43% of posttransplant Burkitt lymphomas and 60% of EBV-negative posttransplant Burkitt lymphomas [7]. Though only a limited number of cases have been reported, several unique clinical features have emerged as helpful diagnostic considerations. BLL-11q mostly affects children and adults younger than age 40 with a male predominance, though cases have been reported in patients up to 82 years old [2, 6–9]. Compared to conventional Burkitt lymphoma, BLL-11q usually has a nodal presentation, often with a single bulky tumor, along with some localized lymphadenopathy [5, 10–12]. The most commonly involved regions are the head and neck and abdominal lymph nodes [10]. Bone marrow or cerebrospinal fluid (CSF) involvement is not typically seen in these cases, though bone marrow involvement has been reported [11]. Rare cases have also been reported in the posttransplant setting, though its role in other immunodeficient states remains unknown [7]. Patients with BLL-11q are usually HIV and EBV negative [13].

### 3.2. Microscopic Features

By morphology, Burkitt-like lymphoma with 11q aberration shares similar features with Burkitt lymphoma, validating the utility of keeping this rare entity on the differential diagnosis of these lesions. Sections show a diffuse proliferation of intermediate-to-large sized cells with round nuclei, small nucleoli, and basophilic cytoplasm accompanied by frequent mitotic figures and apoptotic bodies [9–12]. Sporadically, a follicular or nodular pattern can be observed. The presence of tingible-body macrophages can impart a “starry sky” pattern typically seen in BL. In comparison with BL, however, BLL-11q can demonstrate more cytologic pleomorphism and reduced numbers of macrophages and apoptotic bodies, raising the differential of diffuse large B-cell lymphoma and high-grade B-cell lymphoma [2, 10, 14].

### 3.3. Ancillary Studies

#### 3.3.1. Immunohistochemistry

Providing additional diagnostic complexity, the immunohistochemistry of BL-11q mirrors that of conventional, *MYC-*positive BL with a germinal center (GC) phenotype. All cases show positive expression of CD20, CD10, and BCL-6 with negative expression of MUM1/IRF4 [14]. Most cases in the literature also demonstrate a very high Ki-67 proliferation index (greater than 95%) and negativity for BCL-2, both features that can be seen in BL [10]. To provide assistance in distinguishing between these two entities, several studies have evaluated additional immunohistochemical markers. LMO2 is a marker of germinal center origin that is expressed in germinal center-derived lymphomas as well as acute B-lymphoblastic leukemia and acute myeloid leukemia. Interestingly, LMO2 protein expression is usually downregulated in conventional, *MYC*-positive Burkitt lymphoma. In contrast, positive LMO2 expression can be seen in up to 70% of BLL-11q cases, potentially serving as a useful marker to distinguish between these entities [6].

Additionally, the intensity of *MYC* protein expression appears to differ between these two entities, with conventional BL displaying uniform, bright expression while BLL-11q may show weak, focal *MYC* expression [10]. Rymkiewicz et al. [14] mentioned additional potential immunohistochemical markers, such as CD43, CD44, and CD56, that may provide limited utility.

#### 3.3.2. Flow Cytometry

Flow cytometry analysis shows an antigen expression pattern that is, for the most part, similar to Burkitt lymphoma. The neoplastic cells are usually positive for CD45, pan-B-cell markers (CD19, CD20, and CD22), and CD10 with negative expression for CD5 and CD23. Rymkiewicz et al. investigated a comprehensive flow-cytometry-based profile of Burkitt-like lymphoma with 11q aberration that yielded potentially distinguishing results. In their evaluation, BLL-11q had less frequent diminished expression of CD45 and less frequent CD38^higher^ expression, compared to conventional Burkitt lymphoma. Additionally, CD16/CD56 expression was seen in 60% of BLL-11q lymphomas and in none of *MYC*-positive BL [14].

#### 3.3.3. Cytogenetics

The realm of cytogenetic analysis reveals the most distinguishing feature of Burkitt-like lymphoma with 11q aberration, namely, the 11q aberration. In most studies, conventional cytogenetics and FISH analysis demonstrate a lack of *MYC* (8q24) rearrangements, including the conventional t(8;14), as well as rearrangements in *BCL2* or *BCL6*. All cases demonstrate an abnormality of the 11q arm, with the prototypical abnormality showing high level gains including 11q23.2-23.3 and telomeric losses of 11q24.1-qter. Additionally, compared to conventional BL, BLL-11q cases are more likely to demonstrate complex karyotypes [2, 10, 14]. Gonzalez-Farre et al. reported additional differences from Burkitt lymphoma, namely, the presence of 5q21.3-q32 gains and 6q12.1-q21 losses in BLL-11q and the absence of 1q gains typically seen in BL.9. Despite its utility, two studies state that the 11q aberration pattern is not specifically associated with BLL-11q but may be seen in a subset of cases of *MYC*-positive BL and high-grade B-cell lymphoma (HGBL), NOS [2, 10]. Additionally, gains/amplifications of 11q22-11q24 can be seen in 8–15% of diffuse large B-cell lymphoma (DLBCL) and GC-derived *IRF4*-translocation-positive lymphomas [2]. Therefore, assessment of morphology and immunophenotype remains paramount for recognition of this rare entity.

#### 3.3.4. Molecular Pathology

In BLL-11q, duplication and deletion of the 11q23 and 11q24-qter regions, respectively, simultaneously upregulate and downregulate different sets of genes [2, 11, 13]. Through comparison of gene expression profiles, Salaverria et al. identified two candidate genes involved in the proliferation of the tumor cells: *PAFAH1B2* and *ETS1*. *PAFAH1B2*, which was also upregulated at the protein level, appears to be selectively upregulated in BLL-11q cases, confirming its role as an oncogene in lymphomagenesis. In the region of loss at 11q24-qter, mutations in *ETS1*, a tumor suppressor gene, were identified [2]. A subsequent study echoed these findings, identifying recurrent mutations in *BTG2*, *DDX3X*, *ETS1*, *EP300*, and *GNA13*. Notably, all cases lacked the typical Burkitt lymphoma mutations in the *ID3*, *TCF3*, or *CCND3* genes [10].

### 3.4. Differential Diagnosis

Based on the histologic patterns described above, BLL-11q can mimic a variety of lymphomas. Differential diagnostic considerations for BLL-11q include conventional Burkitt lymphoma, diffuse large B-cell lymphoma, and high-grade B-cell lymphoma (HGBL) [8, 9, 13].

The main differential diagnosis for BLL-11q is conventional, *MYC*-positive Burkitt lymphoma, an entity with which it shares similar morphologic and immunophenotypic features [1, 2]. BL occurs in three distinct clinical settings: endemic, sporadic, and immunodeficiency-associated. These three types exhibit different levels of EBV positivity (predominantly seen in the endemic type) [15, 16], a feature not seen in cases of BLL-11q which are uniformly EBV-negative [13, 14]. Additional distinguishing clinical features of BL include its wider age range, more common extranodal presentation, and more frequent occurrence of bone marrow and CSF involvement [13]. The morphology of BL is well defined, classically displaying a diffuse proliferation of medium-sized lymphocytes with abundant mitotic figures and tingible-body macrophages, imparting the “starry sky pattern” [13, 16]. BLL-11q shares many of these morphologic features; however, it can exhibit more prominent cytologic pleomorphism [10].

The immunophenotype of these two entities is similar, composed of neoplastic B cells with a GC phenotype, negative BCL-2 expression, and a markedly increased Ki-67 proliferative index (approaching 100%) [2, 10]. As mentioned above, markers such as LMO2 and MYC have been proposed as diagnostic stains to separate BL and BLL-11q, with BLL-11q demonstrating positivity for LMO2 and weaker, more focal *MYC* expression [6]. Flow cytometry analysis demonstrates a similar immunophenotype between BL and BLL-11q, with one article highlighting less frequent diminished expression of CD45 and less frequent CD38^higher^ expression in BLL-11q, compared to conventional Burkitt lymphoma [14].

Cytogenetic and FISH analyses allow more definitive classification. More than 90% of BL cases show rearrangements of the *MYC* oncogene, located on chromosome 8. Usually, this rearrangement involves the immunoglobulin heavy chain locus on chromosome 14; however, in up to 20–25% of cases, *MYC* rearranges with the kappa (chromosome 2) or lambda (chromosome 22) light chain loci [1]. Though a subset of conventional Burkitt lymphoma cases can have the defined 11q aberration, these cases nonetheless have the *MYC* rearrangement. BLL-11q, in contrast, demonstrates no *MYC* rearrangements and also lacks additional cytogenetic features reminiscent of BL (1q gains). Thus, cases with suspected Burkitt lymphoma by morphology and immunophenotype that lack *MYC* rearrangements should be worked up for BLL-11q. As mentioned before, molecular analysis is dissimilar in these two entities, demonstrating recurrent mutations in *BTG2*, *DDX3X*, *ETS1*, *EP300*, and *GNA13* for BLL-11q and mutations in the *ID3*, *TCF3*, *and CCND3* genes for BL [10, 11].

Diffuse large B-cell lymphoma (DLBCL) is another diagnostic consideration in this context. Typically occurring in older adults, DLBCL can nonetheless exhibit heterogeneous morphology that may overlap with cases of BLL-11q. Clinically, these patients exhibit nodal or extranodal presentation that can be localized or widespread. Essentially a diagnosis of exclusion, the immunohistochemical profile of DLBCL is positive for pan-B-cell markers (CD19, CD20, and CD22) with variable expression of CD10, BCL-6, and MUM-1, depending on the cell of origin [13, 17]. BCL-2 can be negative in some cases; however, diffuse positivity helps to distinguish DLBCL from BL and BLL-11q. The cytogenetic profile is similarly heterogeneous, with some cases displaying *MYC* rearrangements and an 11q abnormality seen in 8–15% of cases [2]. Nevertheless, the region of gain extends to the telomere and is not coincident with the minimal observed region of gain in BLL-11q [11].

High-grade B-cell lymphoma, similar to BLL-11q, is a new diagnostic entity in the WHO classification system. Replacing the previous category of B-cell lymphoma, unclassifiable, with features intermediate between diffuse large B-cell lymphoma and Burkitt lymphoma (BCLU), this category encompasses a heterogeneous group of aggressive mature B-cell lymphomas. Clinically, these cases are distinct from BLL-11q, usually presenting as advanced, disseminated disease in elderly patients. The morphology of HGBL can display Burkitt-like features but with more cytologic atypia or blastoid morphology. The immunophenotype, like DLBCL, is variable with positive expression of pan-B-cell markers. This entity is in large part defined by its cytogenetic data. The presence of a *MYC* rearrangement, along with *BCL2* and/or *BCL6* rearrangements, is diagnostic of high-grade B-cell lymphoma with *MYC*, *BCL2,* and/or *BCL6* rearrangements (so-called “double/triple hit” lymphoma). HGBL, NOS is a diagnosis of exclusion used very rarely to denote cases lacking the just mentioned cytogenetic findings but with morphology overlapping between DLBCL and BL. HGBL, NOS can demonstrate a *MYC* rearrangement in 20–35% of cases, and a subset has been identified with an 11q aberration. These cases are important to identify due to their unique prognosis and treatment strategies; however, clinical and cytogenetic data help to distinguish it from Burkitt-like lymphoma with 11q aberration [13, 17].

Interestingly, in their assessment of BLL-11q cases, Gonzalez-Farre et al. identified two cases displaying a follicular growth pattern with an underlying meshwork of follicular dendritic cells, raising the differential diagnosis of large B-cell lymphoma with *IRF4* rearrangement. However, these cases did not express IRF4/MUM1 and exhibited the “starry sky” pattern with frequent mitotic figures, a feature not associated with large B-cell lymphoma with IRF4 rearrangement. In summary, a combination of clinical, morphologic, immunophenotypic, and cytogenetic information is needed to fully evaluate and separate these diagnostic entities [10].

### 3.5. Treatment and Prognosis

Currently, the treatment of choice for this tumor is variable and depends on the clinical scenario. Rymkiewicz et al. treated their 10 BLL-11q patients with either a modified R-CODOX-M/R-IVAC regimen (rituximab, fractionated cyclophosphamide, vincristine, doxorubicin, and high-dose methotrexate alternating with fractionated ifosfamide, etoposide, and high-dose cytarabine, along with intrathecal methotrexate and cytarabine) or the GMALL-B-ALL/NHL2002 protocol (rituximab, fractionated cyclophosphamide (or ifosfamide), vincristine, methotrexate, cytarabine, teniposide, and prednisone or doxorubicin). At the last follow-up, 8 of 10 patients were alive (median follow-up = 54 months). Previous cohort studies mentioned in this article suggest that the BLL-11q patients treated with a BL-directed regimen show a similar relapse-free outcome to BL patients. However, BLL-11q patients treated with the conventional DLBCL regimen (R-CHOP) have a higher risk of relapse [14]. Salaverria et al. followed up with their patients for 36 months, and all but one was alive at the last follow-up, suggesting an excellent survival rate for these patients. In general, the five-year overall survival of BLL-11q patients is approximately 80% which is similar to BL. Patients with localized nodal disease appear to show a more favorable outcome after therapy [2]. The treatment of choice in our patient, including induction chemotherapy with cyclophosphamide, vincristine, prednisone, doxorubicin, and methotrexate (COPADM) and consolidation chemotherapy with cytarabine and methotrexate (CYM), was initially introduced for advanced childhood B-cell non-Hodgkin lymphomas and acute lymphoblastic leukemia of the L3 subtype, with similar patient populations being studied in the current ANHL 1131 protocol [18]. This regimen is similar to NCCN-recommended chemotherapy regimens for Burkitt lymphoma, a treatment course that previous articles suggest is preferable to a conventional DLBCL-based regimen [14]. The patient has had a near complete response over the course of treatment.

In conclusion, BLL-11q is a rare and new provisional diagnostic entity with overlapping morphologic and immunophenotypic features with BL. However, unique clinical, cytogenetic, and molecular data can assist in formulating this diagnosis. Therefore, incorporation of multiple ancillary studies may be warranted. Though data remain limited, a supplementary workup for this rare diagnosis is warranted for any case with BL, DLBCL, or HGBCL morphology, germinal center (GC) expression, and a markedly increased proliferative index (>90%), without *MYC* rearrangements. Although there is no definite treatment choice for this lymphoma, an accurate diagnosis is essential in order to choose the most suitable treatment based on the available regimens and clinical trials.
